# Pharmacological and therapeutic directions in ADHD: Specificity in the PFC

**DOI:** 10.1186/1744-9081-4-12

**Published:** 2008-02-28

**Authors:** Florence Levy

**Affiliations:** 1 School of Psychiatry, University of New South Wales, Prince of Wales Hospital, Sydney, NSW 2031, Australia

## Abstract

**Background:**

Recent directions in the treatment of ADHD have involved both a broadening of pharmacological perspectives to include nor-adrenergic as well as dopaminergic agents. A review of animal and human studies of pharmacological and therapeutic directions in ADHD suggests that the D1 receptor is a specific site for dopaminergic regulation of the PFC, but optimal levels of dopamine (DA) are required for beneficial effects on working memory. Animal and human studies indicate that the alpha-2A receptor is also important for prefrontal regulation, leaving open the question of the relative importance of these receptor sites. The therapeutic effects of ADHD medications in the prefrontal cortex have focused attention on the development of working memory capacity in ADHD.

**Hypothesis:**

The actions of dopaminergic vs noradrenergic agents, currently available for the treatment of ADHD have overlapping, but different actions in the prefrontal cortex (PFC) and subcortical centers. While stimulants act on D1 receptors in the dorsolateral prefrontal cortex, they also have effects on D2 receptors in the corpus striatum and may also have serotonergic effects at orbitofrontal areas. At therapeutic levels, dopamine (DA) stimulation (through DAT transporter inhibition) decreases noise level acting on subcortical D2 receptors, while NE stimulation (through alpha-2A agonists) increases signal by acting preferentially in the PFC possibly on DAD1 receptors. On the other hand, alpha-2A noradrenergic transmission is more limited to the prefrontal cortex (PFC), and thus less likely to have motor or stereotypic side effects, while alpha-2B and alpha-2C agonists may have wider cortical effects. The data suggest a possible hierarchy of specificity in the current medications used in the treatment of ADHD, with guanfacine likely to be most specific for the treatment of prefrontal attentional and working memory deficits. Stimulants may have broader effects on both vigilance and motor impulsivity, depending on dose levels, while atomoxetine may have effects on attention, anxiety, social affect, and sedation via noradrenergic transmission.

**Tests of the hypothesis:**

At a theoretical level, the advent of possible specific alpha-2A noradrenergic therapies has posed the question of the role of working memory in ADHD. Head to head comparisons of stimulant and noradrenergic alpha-2A, alpha-2B and alpha-2C agonists, utilizing vigilance and affective measures should help to clarify pharmacological and therapeutic differences.

## Background

Recent directions in the treatment of ADHD have involved both a broadening of pharmacological perspectives to include nor-adrenergic as well as dopaminergic agents. This offers an opportunity, in conjunction with animal studies for a better understanding of the differential selectivity of these agents in the treatment of ADHD. A number of theories have been proposed for the effect of CNS stimulants on dopaminergic (DA) transmission in ADHD.

## Theories of cognitive processing

Moghaddam and Homayoun [1] have described two distinct patterns of cerebral cognitive processing. A modular view is supported by animal and neurophysiological findings [2-4]. A second connectionist view emphasizes parallel distributed neural networks [5-7]. Contemporary approaches consider both types of processing relevant and generally assume that modular processing is a modification of parallel computational networks. The dorsolateral PFC (dlPFC) receives extensive innervations from the mediodorsal nucleus of the thalamus and sends prominent projections to dorsal striatum, nucleus accumbens and ventral tegmental area, allowing a key role in executive functions. On the other hand the orbitofrontal cortex (OFC) receives inputs from sensory associative cortices, particularly olfactory, gustatory, and visual areas, as well as hypothalamus and amygdala.

According to Volkow *et al*. [8], the processing of emotionally salient and behaviourally adaptive information may be at the core of response-reinforcement relations. The role of the frontal cortex, and specifically the anterior cingulate gyrus (CG), in emotional processing has been demonstrated in several imaging studies. Studies indicate an important integrative role for the OFC and CG in the analysis of information that carries an emotive, evaluative and in the long term, survival significance for an individual [8].

## Synaptic DA transmission

Seeman and Madras [9] suggested that methylphenidate (MP) blocks the dopamine transporter (DAT), resulting in increased extracellular DA, activating autoreceptors and leading to an attenuation of DA release in response to phasic DA firing. On the other hand, a second hypothesis by Volkow *et al *suggests that the blocked DAT overcomes the inhibitory effects for activation of the autoreceptors, leading to a net effect of DA accumulation in the synapse, with amplification of DA signals resulting from tonic as well as phasic DA [8].

Grace has pointed out that by interfering with dopamine re-uptake, stimulants allow dopamine to escape the synaptic cleft, thereby depressing subsequent spike-dependent phasic dopamine release by increasing the tonic stimulation of the auto-receptor. Thus subcortical down-regulation depends on presynaptic auto-inhibition through autoreceptors [10,11]. This mechanism is similar to that proposed by Seeman and Madras, who pointed out that stimulants raise extracellular levels of dopamine several-fold, but reduce the extent to which dopamine is released with nerve impulses, compared with the impulse-associated release in the absence of the drug. However at higher doses, stimulants are found to produce generalised stimulation of the nervous system, as a result of very high concentrations of extracellular dopamine at rest, and markedly increased release of dopamine with nerve impulses overcoming presynaptic inhibition of dopamine release [12].

Volkow *et al*. used positron emission tomography (PET) to demonstrate that extracellular DA increases in proportion to the level of DAT blockade, and the rate of DA release by cell firing. The latter increases were found to be greater when methylphenidate was given concomitantly with a salient rather than a neutral stimulus. The authors postulated that enhanced saliency and MP motivates the improved school performance observed with methylphenidate [8]. According to Volkow *et al*., symptoms of inattention have been mainly linked with striatum and cingulate gyrus, those of hyperactivity with striatum, and those of impulsivity with nucleus accumbens, while impairment in executive tasks is linked with the dorsolateral prefrontal cortex [13,14].

## Dopamine and working memory

Goldman-Rakic *et al*. investigated the pharmacological actions of drugs on neurons as they are engaged in cognitive processes in awake behaving animals [15]. They have shown that the 'memory fields' of the prefrontal cortex are modulated by neurotransmitters such as dopamine serotonin and gamma-amino-butyric acid (GABA) in different ways [16-18]. Goldman-Rakic *et al*. showed that the spatial tuning of prefrontal neurons engaged in spatial working memory was enhanced at moderate levels of occupancy, and reduced at both lower and higher levels of occupancy. The specificity of drug action on single cell activity was thought accounted for by the specific synaptic arrangement of D_1 _receptors in spines that receive excitatory inputs from visual pathways carrying highly processed visuo-spatial information. The model postulated an optimal level of D_1_receptor occupancy for efficient physiological signalling and optimal performance [15].

Spatial working memory has been associated with the dorsolateral PFC, while the orbital and ventromedial PFC allow recognition and inhibition of emotional responses, important for appropriate social behaviour [19]. Goldman-Rakic and colleagues pointed out that a full understanding of the functional capacity of even a single pyramidal cell requires knowledge not only of its biophysical properties but its circuitry and signalling mechanisms in vivo. Thus experimental depletion of dopamine in prefrontal areas of rhesus monkeys has been shown to produce impairments in working memory performance [15,20]. Furthermore Goldman-Rakic *et al*. [21] and Lidow *et al*. [22] have showed that the D_1 _family of dopamine receptors are at least 20-fold more abundant than D_2 _family of receptors in the PFC. The D_1 _receptor was also found to be in close proximity to putative glutamatergic axon terminals, giving rise asymmetric synapses on the same spine.

Muly *et al*. also studied the distribution of D_1 _receptor in GABAergic interneurons, and shown it is preferentially found in those subtypes of interneurons which provide the strongest inhibitory input to the perisomatic region of cortical pyramidal cells [23]. They suggested a circuit model to explain the biphasic action of D_1 _receptor stimulation on working memory performance and neuronal delay period firing, which focuses on the interactions between pyramidal and non-pyramidal neurons. The authors believe that D_1 _receptor stimulation enhances excitatory transmission to both pyramidal cells and interneurons, but the enhancement is more effective on pyramidal cells, because of the closer contacts with dopaminergic axon terminals. On pyramidal neurons, the spine may act as a diffusion barrier, while on interneurons D_1 _receptor synapses are located on the shaft allowing for more diffusion of second messengers [24,25].

## COMT and development

In humans, the catechol-O-methyltransferase (COMT) gene contains a common variation in its coding sequence, which translates valine (Val) to methionine (Met). At room temperature the Met allele has one-fourth the enzyme activity of the Val allele. It is believed that COMT is important in the metabolism of DA in the prefrontal cortex (PFC) whereas the dopamine transporter (DAT) is more important in the striatum. The Val allele associated with high -activity COMT increases phasic and reduces tonic DA transmission sub-cortically and decreases DA concentrations cortically. This leads to an increase in D_2 _and a decrease in D_1 _receptor transmission; as a result there is decreased stability of neural networks, underlying working memory representations. The Met allele associated with low-activity COMT decreases phasic and increases tonic DA transmission sub-cortically, and increases DA concentrations cortically: this is associated with increased D_1 _and decreased D_2 _transmission in the PFC [26]. This increases the stability of networks mediating sustained working memory representations. A recent study found a small but significant relationship between Val^158^Met genotype and executive function in healthy individuals but not in schizophrenia [27]. Thus the COMT gene (possibly in conjunction with other DA genes) may help predict DA function in the PFC. However, genetic studies in ADHD have shown limited association with ADHD [28]. This may be a developmental issue related to the late development of cortical PFC connections in adolescence, with late 'turning on' of the COMT gene [29].

According to Lewis and Gonzalez-Burgos, dendritic spines are the principal targets of excitatory synapses to pyramidal neurons [30]. Excitatory connections from the mediodorsal thalamus, the principal source of inputs to the dlPFC, synapse primarily on dendritic spines. Dendritic spine density on dlFPC layer 3 pyramidal neurons undergoes a substantial decline during adolescence in primates. Activity-dependent strengthening and pruning appears to underlie synapse stabilisation. It is suggested that functionally immature synapses may not be able to provide compensation for synaptic dysfunction prior to adolescence, because they have a very low AMPA receptor contribution, rendering them silent at the resting memory potential, and a relatively high probality of glutamate release, and thus subject to quick exhaustion of glutamate pools after repetitive activation. It is likely that a range of factors including labour-delivery, alcohol, nicotine and other stressors may affect the development of dlPFC circuitry. Dopamine is thought to exert a modulating influence on the dlPFC. It is thought that prefrontal pyramidal neurons may directly excite mesocortical DA neurons in the ventral mesencephalon and indirectly inhibit mesotriatal DA cells through activation of GABAergic neurons in mesencephalic cell nuclei [30]. Thus reduction in PFC cell activity leads to an excess of DA receptor activation at subcortical nuclei. In the primate dlPFC, D1 receptors are the most abundant DA receptor subtype, and mediate most of the cellular effects of DA in this subtype, and mediate most of the cellular effects of DA in this region [31]. Thus D1 receptors regulate sustained firing of dlPFC neurons during the delay phase of delayed-response tasks that require working memory [32,33]. DA neurons may also have an important role in gating information loaded into working memory buffers [34-37].

According to Lewis and Gonzalez-Burgos, COMT is mostly an intracellular cystolic protein [38]. Therefore, extracellular DA must be transported through the plasma membrane before becoming a substrate of COMT enzymatic activity, raising the question of potential interaction between genetic variants of COMT and the DA transporter. However, because COMT expression levels in DA neurons are relatively low [39,40], the availability of DA to be inactivated by COMT is dependent on DA transport through the membrane of cortical cells that express higher levels of COMT (e.g. post-synaptic neurons or glia), and that may express non-specific transporters with the ability to uptake DA. It is thus important, according to the authors, to note that COMT metabolises norepinephrine, which heavily innervates the primate dlPFC [41] and influences working memory function [42].

Lewis and Gonzalez-Burgos [30] point out that although the interneuron depolarization and the increase in inhibitory currents produced by DA suggests net inhibitory effects in the primate dlPFC, DA also changes the response of pyramidal neurons to excitatory currents with a delayed time course [32] and is consistent with other delayed D1 receptor-mediated effects [36]. This may partly account for the inverted-U dose-response effect on dlPFC firing, though these effects may be complex [43] Low levels of DA receptor stimulation appear to selectively increase the firing of neurons that are tuned to preferred stimuli during tasks that require working memory [16].

## Developmental hypothesis

While an association has been proposed between DRD1 and schizophrenia, most genetic association studies in ADHD have been directed towards the dopamine transporter, DAT1 and the DRD4 receptor. An influential developmental hypothesis, the Dynamic Developmental Theory (DDT) suggested that the dopaminergic system is hypoactive in ADHD, and explains the behavioural changes in terms of altered reinforcement and extinction processes [44]. This leaves open the question of the role of working memory in ADHD, presumably related to deficits in the prefrontal DRD1 system. The present developmental hypothesis suggests early deficits in PFC connectivity, in both young children and in ADHD. In this case stimulant medications may 'remediate' a deficient working memory system, as suggested in neuropsychological studies of vigilance [45,46].

## Noradrenergic mechanisms

The above 'vigilance/working memory' hypothesis also draws attention to the noradrenergic (NA) system. Optimal prefrontal cortex function is thought to require optimally functioning noradrenergic and dopaminergic input to the prefrontal cortex [47,48]. A relatively recent approach to the treatment of ADHD has been the finding of beneficial effects from alpha-noradrenergic agents such as atomoxetine and guanfacine. The molecular genetic basis for the use of noradrenergic agents has been best reviewed by Arnsten *et al*. [49]. They point out that it has been appreciated for some time that dopamine (DA) is necessary for proper prefrontal cortex (PFC) function, but more recently a role for noradrenergic transmission has been appreciated. According to Arnsten *et al*., the role of the D_1 _receptor family in the regulation of PFC function has received particular focus, particularly as D_1 _receptors are found in high concentration in the PFC on dendritic spines. This allows DA to modulate incoming information to PFC pyramidal cells [50,51]. On the other hand, supra-normal DA stimulation may impair PFC function, providing an inverted-U effect. For example, Goldman-Rakic *et al*., found that the D1 message and protein in the cortex of rhesus monkeys was down-regulated by chronic neuroleptic treatment, which both down-regulated D1 receptors and produced impairments in working memory performance [23].

According to Arnsten *et al*. electrophysiological recordings of PFC neurons in monkeys performing working memory tasks, have shown that PFC neurons are able to hold modality-specific information "on-line" over a delay period and use this representational information to guide behaviour in the absence of environmental cues. The delay-related firing is thought to arise from feed-forward microcircuits within the PFC, as cells with shared properties interconnect [52]. Arnsten *et al*. suggest there are no drugs that distinguish between D_1 _and D_5 _receptors, and moderate levels of D_1_/D_5 _receptor stimulation suppress delay-related firing for non-preferred spatial directions, and thus enhance spatial tuning. The authors point out that while limited studies have been done in humans, due to the lack of selective D_1_/D_5 _compounds available for human use, studies with non-selective compounds suggest that an "inverted-U" response may be evident in humans as well as animals. They suggest that compounds that prefer D_1 _receptors may be more helpful than D_2 _agonists in improving verbal memory [52].

## Alpha-2A receptor

According to Arnsten [53], relatively high doses of alpha-2 agonists appear to have beneficial effects on cognitive function, although these effects may be eroded by emerging sedative and hypotensive effects. However, Arnsten *et al*. [52] and Arnsten and Leslie [54] have shown that the ability of alpha-2 agonists to improve PFC function without side effects was found to correspond with selectivity for the alpha-2A receptor site. A comparison between the three NA alpha-2 receptors guanfacine, clonidine and BHT showed guanfacine had better selectivity than clonidine, which is more selective than BHT 920. Thus open trials [55-57] have shown beneficial effects of guanfacine, including improved performance on the Continuous Performance Task [58]. Arnsten suggests that NA-alpha-1 and NA-alpha-2 receptors may have opposing effects in the PFC, and that alpha-2 mechanisms may predominate when basal NA release is moderate, as in normal attentive wakening, and alpha-1 mechanisms may predominate under conditions of stress with higher levels of NA release. Thus alpha-2 agonists such as guanfacine may protect PFC cognitive function during stress by preventing excessive NA or DA release in the PFC [53].

Easton *et al*. utilised phMRI BOLD (magnetic resonance imaging) contrast to image the blood oxygenation level dependant response in rat brain regions following administration of guanfacine [59]. They postulated that activation of alpha-2 receptors in the dorsolateral PFC by an agonist such as guanfacine might facilitate PFC neuronal activity and in turn exert an inhibitory influence on other cortical areas (such as premotor and motor areas) and/or subcortical structures (such as the striatum) that are involved in the control of locomotion. Time-course analyses of saline vs guanfacine effects were carried out, using both fixed and random effect analyses. Random effect analysis showed that guanfacine produced positive BOLD responses in frontal areas, including the frontal association and secondary motor cortex and prelimbic region of the PFC. This is consistent with single-photon emission computed tomography (SPECT) evidence, which shows improved cognitive performance and increased rCBF (cerebral blood flow) values in the dorsolateral PFC following guanfacine administration to young adult rhesus monkeys, and with human positron emission tomography (PET) data showing increased regional cerebral blood flow in frontal lobes following guanfacine administration. Positive BOLD changes also occurred in the dentate gyrus and CA_1. _Guanfacine produced widespread negative BOLD effects in the caudate, putamen, nucleus accumbens and entorhinal cortex, suggesting decreased dopaminergic neuronal function in this area.

Easton *et al*. interpreted their data as suggesting that guanfacine acts on the prefrontal cortex (probably post-synaptically at alpha- 2 receptors) to increase cognitive and associated functions, known to be dysfunctional in ADHD sufferers, and also helps in the regulation of locomotor activity via inhibitory control of subcortical brain regions, particularly the caudate putamen and nucleus accumbens [59]. Thus guanfacine appeared to have the ability to 'turn down' striatal activity, possibly of benefit in the treatment of motoric hyperactivity. The investigators also demonstrated an intense positive BOLD effect in the lateral hypothalamic area, which is strongly associated with feeding behaviour, perhaps a basis of appetite or gastrointestinal side effects [60,61].

According to Easton *et al*. the alpha-2A adrenergic receptor subtype appears to be the site of action of the beneficial clinical effects of alpha-2A agonists on the prefrontal cortex (PFC) [59]. While these receptors are localized both pre- and post- synaptically, some lines of evidence are thought to suggest that their site of action is post-synaptic in the PFC [62]. Thus, guanfacine has been shown to dose-dependently prevent deficits of spatial working memory, suggesting a role in cognitive deficits associated with NMDA receptor hypofunction [63,64]. Application of the D_1 _receptor agonist SKF81297 has been shown to cause a prominent increase of steady-state NMDA-evoked current in acutely isolated PFC pyramidal neurons, and up-regulation of NMDA receptor activity by dopamine D_1 _receptors suggests reciprocal interactions between D_1 _and NMDA receptors [65].

Arnsten *et al*. suggest that stimulants act to enhance the release of and/or inhibit the reuptake of both DA and NE (norepinephrine) [54]. Arnsten and Dudley [66] have shown that the PFC-enhancing effects of methylphenidate are prevented by blockade of either NE alpha-2 or DAD1 receptors, suggesting that stimulants facilitate endogenous stimulation of D_1 _and alpha-2A receptors in the PFC. It is thought to increase delay-related firing and strengthens the functional connectivity of microcircuits in the PFC. In children, Scahill *et al*. found that immediate release guanfacine was rated significantly better than placebo by teacher-rated ADHD (37% vs 8%) and subjects performed significantly better on a Continuous Performance Test [67].

Arnsten *et al*. also suggest that as with DA, moderate levels of NE are critical for proper PFC function [54]. They suggest that the majority of alpha-2 receptors are localised postsynaptically to NE terminals, and that blockade of alpha-2 receptors in the PFC of monkeys erodes delay-related cell-firing and recreates all the symptoms of ADHD, with poor impulse control, impaired working memory with underlying distractibility.

Wang *et al*. have shown that spatial working memory is maintained by spatially tuned recurrent excitation within networks of prefrontal cortical neurons [68]. They investigated monkeys performing on an oculomotor spatial delayed response, which required monkeys to make a memory-guided saccade to a visuo-spatial target. Neurons recorded from area 46 of the dorsolateral PFC were isolated and subjected to iontophonetic application of pharmacological agents. Intra-PFC administration of guanfacine was shown to significantly enhance delay-related activity for the 180° location (preferred direction) in 28 out of 35 cases. Studies indicated that suppression of cyclic AMP impaired WM performance.

Wang *et al *suggest that cAMP (cyclic AMP) has powerful influences on Hyperpolarisation Activated Cyclic Nucleotide-gated (HCN) channels that pass on h current when opened [68]. They are localised on distal pyramidal dendrites and according to the authors, are co-expressed with the alpha-2A adrenoreceptor, thus providing a potent substratum for functional integration in the primate PFC. In electrophysiological studies with alpha-2A adrenoreceptor stimulation or cAMP inhibition, HCN channel blockade enhanced spatially tuned delay-related firing of PFC neurons. Exposure to uncontrollable stress via excessive catecholamine release, high levels of D_1 _receptor stimulation, or by activating cAMP, has been shown to impair working memory. Under these conditions, the PFC is functionally disconnected rendering it "decorticate". The process may be exacerbated in patients with aberrant genes that regulate cAMP signaling e.g. COMT [69]. This co-localisation of cAMP, HCN channels and D_1 _excess stimulation effects may help to explain the cross-talk between DA and NE receptors in the PFC discussed below.

Vijayraghavan *et al*. showed that dopamine D_1 _receptor stimulation in PFC produced an 'inverted-U' dose-response, whereby either too little or too much D_1 _receptor stimulation impaired spatial working memory [69]. This response has been observed across species, including genetic linkages with human cognitive abilities, PFC activation states and DA synthesis. According to the authors the cellular basis for the inverted U has long been sought, with in vitro intracellular recordings supporting a variety of potential mechanisms. Their study demonstrated that the D_1_receptor agonist inverted-U response was observed in PFC neurons of behaving monkeys: low levels of D_1 _receptor stimulation enhanced spatial tuning by suppressing responses to non-preferred directions, whereas high levels reduced delay-related firing for all directions, eroding tuning. These actions of D_1 _receptor stimulation were mediated in monkeys and rats by cyclic AMP intracellular signaling. The evidence for an inverted-U at the cellular level in behaving animals promised to bridge in vitro molecular analyses with human cognitive experience.

Arnsten *et al*. have described three different subtypes of alpha-2 adrenoreceptors in humans, the alpha-2A alpha-2B and alpha-2C subtypes [54]. The alpha-2A and alpha-2C subtypes are widely distributed in the brain, including the PFC, the alpha-2B receptor is most concentrated in the thalamus. Guanfacine is thought to be the most selective agonist available for the alpha-2A subtype [70]. On the other hand the sedating effects of clonidine are thought to involve the thalamus, basal forebrain and other alpha-2B and alpha-2C effects. Atomoxetine, which also has sedating effects in some children, may also have alpha-2B and/or alpha-2C effects [48].

A recent study by Chamberlain *et al*. suggested that NE is more sensitive in modulating lateral compared to orbital PFC functioning [71]. They showed that atomoxetine (60 mg single dose) improved response inhibition on a stop- signal task, but had no effect on a task requiring probabilistic learning. On the other hand administration of citalopram (30 mg single dose) impaired probabilistic learning with no effect on response inhibition. The authors concluded that finding that atomoxetine improved response inhibition in healthy volunteers implicated ascending noradrenergic systems in its control, whereas the role of 5-HT in probabilistic learning might operate according to an inverted-U function.

## Clinical implications

While clinical studies of guanfacine are limited, despite some promising results, there has been considerable interest in the use of atomoxetine. Despite some encouraging trial results, [72] anecdotal clinical reports suggest limited success in some children, and there have also be concerns about suicidal ideation in a small number of patients [73]. This raises the question of the pharmacological actions of atomoxetine, which also increases extrasynaptic levels of NE and DA in the PFC.

The selectivity of atomoxetine for NE effects, was investigated by Bymaster *et al*., utilising radioligands and microdialysis studies [74]. They showed that atomoxetine increased extracellular levels of NE in the prefrontal cortex 3-fold, but did not alter 5-HT levels. Atomoxetine also increased DA concentrations in the PFC 3-fold but did not alter DA in nucleus accumbens or striatum. In contrast methylphenidate increased DA and NE equally in PFC, but also increased DA in striatum and nucleus accumbens to the same level. The authors suggest that atomoxetine is thus less likely to have motoric or abuse liability.

Arnsten and Li suggest that noradrenergic transmission has a vital beneficial effect on PFC function, particularly via postsynaptic alpha-2-adrenoreceptors. Alpha -2 agonists such as guanfacine have been shown to improve working memory performance in monkeys after direct infusion into the dorsolateral PFC [75]. Blockade of alpha -2 receptors with yohimbine infusion was shown to weaken working memory [76], and in addition to induce motor hyperactivity [77]. Arnsten and Li have pointed out that the PFC guides behaviour, thought and affect using working memory, and that these processes are the basis of executive functions, including regulation of attention, planning and impulse control [75]. PFC function is thought to be fundamental in ADHD [48,49]. As outlined above, stimulation of the D_1_receptor within optimal levels improves working memory performance, while higher levels may erode performance [78,79]. The role of D_2_, D_3 _and D_4 _receptors in the PFC are, according to Arnsten and Li less well understood, though it is of interest that NA has high affinity for the D_4 _receptor, which may be considered a catecholamine rather than a DA receptor [75].

Interestingly, while it has been accepted in the past that the beneficial effect effects of stimulant medication were mediated by DA transmission, Arnsten and Li suggest that performance on PFC tasks in rats and mice on low oral doses of methylphenidate may be mediated in part by the alpha-2 adrenoreceptor [75]. Thus stimulants, atomoxetine and guanfacine may exert therapeutic actions via D_1 _and alpha-2A receptor mechanisms, but under conditions of stress, guanfacine may be more protective [80], given that high doses of stimulants may impair cognition and induce perseverative or restricted thinking [81].

## Receptor specificity

Arnsten describes the PFC as using representational knowledge, i.e. to guide overt responses as well as allowing inhibition of inappropriate behaviours by gating the processing of irrelevant stimuli. Studies in rats have shown that low oral doses of methylphenidate that produce plasma drug levels similar to therapeutic doses in humans, also improve PFC function, dependant on both dopamine D_1 _and NE- alpha-2 receptor stimulation [82].

Acccording to Arnsten, the enhancing effects of alpha-2A agonists are most likely mediated through G-mediated suppression of CAMP intracellular signaling. Blocking alpha-2 receptors in monkey PFC with yohimbine reduces delay-related cell firing and impairs working memory, as well as impulse control, recreating the profile of ADHD. In contrast excessive stimulants produce cognitive inflexibility through alpha 1, beta and high D1 stimulation [66].

It is still somewhat unclear whether DA effects in the PFC are exerted primarily through the DAD1 receptor, or alpha-2A receptor. Cornil *et al *have shown that dopamine activates noradrenergic receptors in the quail preoptic area [83]. They found that DA-induced inhibitions/excitations were not blocked by selective dopaminergic receptor antagonists, but were suppressed by selective alpha-noradrenergic antagonists (yohimbine/prasozin). While the mechanism of the cross-talk between DA and NE receptors was unclear, the relatively similar structure of DA and NE could potentially explain the binding of both amines to the two receptor subtypes, as could co-localisation of cAMP, HCN channels and alpha-2A adrenoreceptor [68]. Studies of transgenic mice demonstrated that the transporters for NE and DA lacked specificity, so that in the prefrontal cortex, DA was mainly taken up by the NE transporter [84]. The mechanisms and specificity in human therapeutics remains an intriguing problem whose solution offers more targeted understanding of future treatments.

## Conclusion

The above understandings have given rise to newer treatment approaches. In particular there has been increased interest in the efficacy of both non-stimulant and long-acting agents. A variety of extended release dopaminergic agents are being or have been produced by pharmaceutical companies but so far none have been selective for the PFC alone, and may also be subject to motor and stereotypic effects at striatal level. Taken together the data seems to suggest differences in specificity in the current medications used in the treatment of ADHD, with guanfacine likely to be most specific for the treatment of attentional deficits, while stimulants may have broader effects on both vigilance and motor impulsivity depending on dose levels. Atomoxetine has effects on attention, social affect, anxiety and sedation via noradrenergic systems. Head to head comparisons utilizing both vigilance and affective measures, such as facial recognition may further elucidate differential effects. At a theoretical level, the advent of specific noradrenergic therapies such as guanfacine, raises the question of the role of working memory in the treatment of ADHD. Also suggested, is a possible aetiological role of developmental deficit or delay, at the prefrontal cortical level, in ADHD.

## Competing interests

The author declares that she has no competing interests.

## Abreviations

D1 Dopamine D1 receptor: PFC Prefrontal cortex; OFC Orbitofrontal cortex;

DAT Dopamine transporter; COMT Catechol-O-methyltransferase;

NA Noradrenergic; NE Norepinephrine;

MRI Magnetic Resonance Imaging; BOLD Blood oxygen level dependent;

SPECT Single-photon emission computed tomography; NMDA N-methyl D-aspartate;

CAMP Cyclic AMP; HCN Hyperpolarisation Activated Cyclic Nucleotide-gated
